# The level of partial pressure of carbon dioxide affects respiratory effort in COVID-19 patients undergoing pressure support ventilation with extracorporeal membrane oxygenation

**DOI:** 10.1186/s12871-023-02382-9

**Published:** 2024-01-12

**Authors:** Yuankai Zhou, Xinchen Wang, Wei Du, Huaiwu He, Xiaoting Wang, Na Cui, Yun Long

**Affiliations:** Department of Critical Care Medicine, State Key Laboratory of Complex Severe and Rare Diseases, Peking Union Medical College, Peking Union Medical College Hospital, Chinese Academy of Medical Sciences, Beijing, 100730 China

**Keywords:** PaCO_2_, COVID-19, Respiratory effort, Extracorporeal membrane oxygenation

## Abstract

**Background:**

Patients with COVID-19 undergoing pressure support ventilation (PSV) with extracorporeal membrane oxygenation (ECMO) commonly had high respiratory drive, which could cause self-inflicted lung injury. The aim of this study was to evaluate the influence of different levels of partial pressure of carbon dioxide(PaCO_2_) on respiratory effort in COVID-19 patients undergoing PSV with ECMO.

**Methods:**

ECMO gas flow was downregulated from baseline (respiratory rate < 25 bpm, peak airway pressure < 25 cm H_2_O, tidal volume < 6 mL/kg, PaCO_2_ < 40 mmHg) until PaCO_2_ increased by 5 − 10 mmHg. The pressure muscle index (PMI) and airway pressure swing during occlusion (ΔPOCC) were used to monitor respiratory effort, and they were measured before and after enforcement of the regulations.

**Results:**

Ten patients with COVID-19 who had undergone ECMO were enrolled in this prospective study. When the PaCO_2_ increased from 36 (36 − 37) to 42 (41–43) mmHg (*p* = 0.0020), there was a significant increase in ΔPOCC [from 5.6 (4.7–8.0) to 11.1 (8.5–13.1) cm H_2_O, *p* = 0.0020] and PMI [from 3.0 ± 1.4 to 6.5 ± 2.1 cm H_2_O, *p* < 0.0001]. Meanwhile, increased inspiratory effort determined by elevated PaCO_2_ levels led to enhancement of tidal volume from 4.1 ± 1.2 mL/kg to 5.3 ± 1.5 mL/kg (*p* = 0.0003) and respiratory rate from 13 ± 2 to 15 ± 2 bpm (*p* = 0.0266). In addition, the increase in PaCO_2_ was linearly correlated with changes in ΔPOCC and PMI (R2 = 0.7293, *p* = 0.0003 and R2 = 0.4105, *p* = 0.0460, respectively).

**Conclusions:**

In patients with COVID-19 undergoing PSV with ECMO, an increase of PaCO_2_ could increase the inspiratory effort.

**Supplementary Information:**

The online version contains supplementary material available at 10.1186/s12871-023-02382-9.

## Background

Excessive respiratory effort may cause self-inflicted lung injury (SILI) and inspiratory muscle injuries [1–3], stimulate desynchronization between the patient and ventilator [4], and worsen the perfusion of extrapulmonary organs [5]. Appropriate respiratory drive and effort should be maintained during the treatment of patients with respiratory failure [6]. In contrast, respiratory drive and effort are commonly increased in patients with COVID-19 pneumonia [7], and this phenomenon may persist in critically ill patients with COVID-19, even after receiving venovenous extracorporeal membrane oxygenation (vv-ECMO) support, owing to low pulmonary compliance and a high systemic inflammatory state [8].

To reduce respiratory effort and drive, physicians often administer high doses of sedative drugs, analgesics, and muscle relaxants. The prolonged use of high doses of these drugs can cause loss of the spontaneous cough reflex, which in turn impairs sputum drainage and eventually worsens pulmonary consolidation and lung infections.

As the partial pressure of carbon dioxide in arterial blood (PaCO_2_) could affect the respiratory drive from the respiratory center [1], it has been shown that altering different levels of extracorporeal carbon dioxide removal in patients undergoing ECMO recovering from acute respiratory distress syndrome (ARDS) could alter respiratory drive [9]. We hope to explore the effect of PaCO_2_ level on respiratory effort in patients with COVID-19 undergoing ECMO.

## Materials and methods

The study was performed based on the Declaration of Helsinki. All experiments were performed in accordance with relevant guidelines and regulations. The ethics committee of Peking Union Medical College Hospital approved this study(Ethics certificate number:K23C1385). Written informed consent was provided from the patients and from the next of kin of all enrolled patients.

### Patient enrollment

The study was conducted in the intensive care unit of Peking Union Medical College Hospital in China. Patients with COVID-19 who had undergone ECMO and pressure support ventilation (PSV) via tracheal intubation between December 2022 and March 2023 were considered eligible for inclusion.

Drainage blood was drained using a 21 Fr cannula and return blood was drained using a 17 Fr cannula to achieve a blood flow of up to 5 L/min. ECMO blood flow was typically 3.0-3.5 L/min, while sweep gas flow (GF) was 3–9 L/min to maintain arterial oxygenation and normocapnia.

On admission, we recorded data on age, sex, predicted body weight, Sequential Organ Failure Assessment (SOFA) score, static respiratory system compliance, arterial blood gas analysis, ventilation analyses, ventilation and ECMO settings (blood flow, GF), and days on ECMO.

### Measurements

Measurement of respiratory effort: 1) Pressure muscle index(PMI): Using the airway occlusion method, we put forward a simple estimate of the pressure developed by the inspiratory muscles at end inspiration. During the pressure support mode, the inspiratory hold button was pressed and a physician performed an end-inspiratory occlusion maneuver. After a certain period, the patient completely stopped inspiratory effort. The difference between the end-inspiratory obstructive plateau pressure and pre-obstructive airway pressure (Paw) was used to estimate the patient’s inspiratory effort and referred to as PMI [10, 11](Figure S1-A). 2) Airway pressure swing during occlusion (ΔPOCC): ΔPOCC is defined as the swing in the Paw generated by the force of the respiratory muscle under assisted ventilation when the airway is temporarily blocked [3]. The expiratory airway occlusion of the ventilator was performed at random intervals during each recording. Each occlusion persisted for a single breath, verified by the Paw recovery to normal. The maximum deviation of Paw from positive end-expiratory pressure (PEEP) during each occlusion was documented as ΔPOCC (Figure S1-B).

All patients were receiving mechanical pressure support ventilation (SV800 Ventilator, Mindray, Shenzhen, China) and monitoring of end tidal carbon dioxide (etCO_2_) (CAPNOSTAT M2501A CO_2_ Sensor, Philips, Netherlands).

### Study protocol

A stable environment was maintained during the study to avoid stress and abrupt stimulation.

Before the start of the study, sedative drugs were titrated to Richmond agitation sedation scale values of − 3 to − 2, an assisted breathing mode trial was conducted, and support pressure level were adjusted to achieve tidal volume < 6 mL/kg. The ECMO GF was adjusted to achieve stable baseline conditions, defined as PaCO_2_ < 40 mmHg, respiratory rate < 25 bpm, and peak airway pressure < 25 cm H_2_O. PEEP, fraction of inspired oxygen, PSV, ECMO blood flow, and dose of norepinephrine, sedatives, and analgesics remained unchanged throughout the study.

**Fig. 1:**
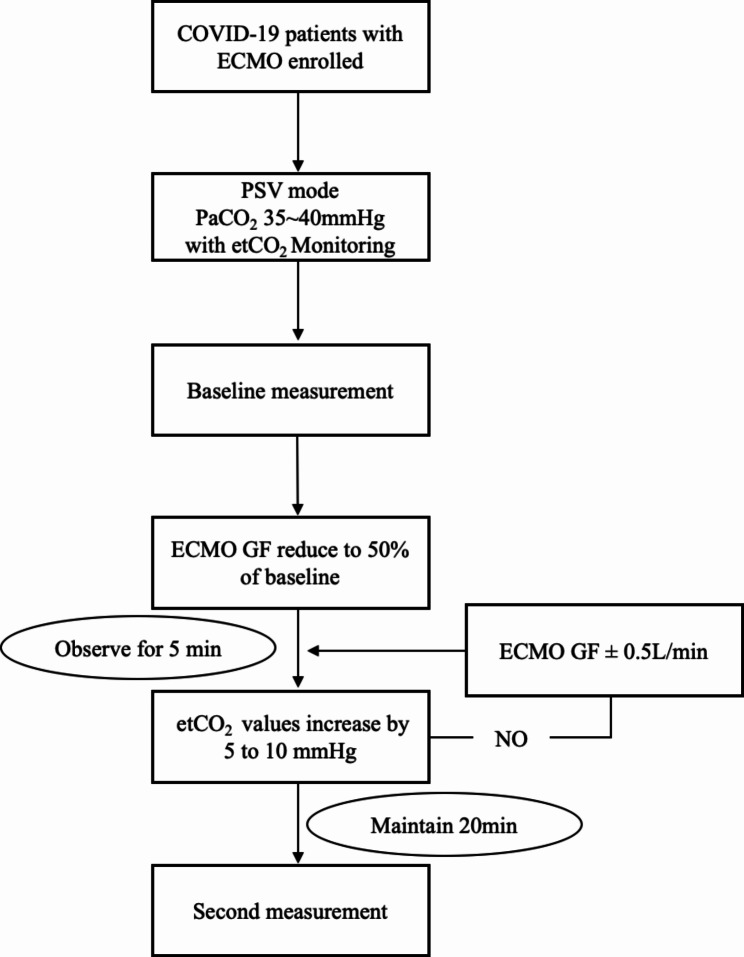
Study protocol. ECMO, extracorporeal membrane oxygenation; PSV, pressure support ventilation; etCO_2_, end tidal carbon dioxide; GF, gas flow

The study protocol was initiated when the baseline parameters were stable. The baseline parameters, including ventilation settings, arterial and arterial blood gas analysis, hemodynamics, and indicators of respiratory effort were measured in the baseline phase. Then, the ECMO GF was modified to 50% of the baseline, and etCO_2_ values were monitored. ECMO GF was adjusted at 5-min intervals (increasing or decreasing by 0.5 L/min each time) until etCO_2_ stabilized at a level 5–10 mmHg higher than the baseline. After 20 min, the parameters were measured for the second time in the high-CO_2_ phase (Fig. 1).

In this study, the primary endpoint parameters were PMI and ΔPOCC, and the secondary endpoint parameters were respiratory parameters such as respiratory rate and tidal volume.

The study was stopped if the heart rate (HR) was > 140 bpm and/or respiratory rate was > 40 bpm and/or systolic blood pressure > 180 mmHg and/or patients experienced anxiety or diaphoresis.

### Statistical analysis

Descriptive analysis was performed. All data are expressed as mean ± standard deviation or the median (25–75%, interquartile range). The Shapiro-Wilk test was used to evaluate normality. Variables were compared between the baseline and high-CO_2_ phase using the Student’s paired t-test or Wilcoxon matched-pairs signed-rank test. Linear correlations were analyzed using the Pearson’s test. In our pre-experiment, we found that the change in PaCO2 doubled ΔPOCC and PMI. The study was designed with 80% power to detect the minimum difference between the two phases, with a two-tailed alpha of 0.05. The calculated sample size was 9. Furthermore, the sample size was similar to that of previous studies [9, 12]. All comparisons were two-tailed, and a *p* < 0.05, was required to exclude the null hypothesis. SPSS version 25.0 (IBM Corp., Armonk, NY, USA) was used for the statistical analysis.

## Results

Ten patients with COVID-19 who had undergone ECMO were enrolled between December 2022 and March 2023. All patients successfully completed the study protocol. Nine patients received vv-ECMO with internal jugular-femoral vein access, while the drainage cannula was femoral venous cannula and the return one was right internal jugular venous catheter. One patient had a double-lumen cannula in the neck. Each patient had two phases of measurement data (baseline and high-CO_2_ phase). Demographic and basic hemodynamic parameters are presented in Table 1. All patients were men and with a mean age of 75 ± 9 years. On admission, the SOFA score was 12 ± 2, the Mechanical Ventilation(MV) time was 22 ± 15 days, and the mean ECMO time was 12 ± 2 days.

**Table 1:** Characteristics of the Ten Spontaneously Breathing COVID-19 Patients with Extracorporeal Membrane Oxygenation Enrolled

Patients Number	Age (year)	Sex	SOFA	Days on ECMO before Enrolment	Days on MV before Enrolment	Cst (ml/cm H_2_O)	PEEP (cm H_2_O)	PSV Level (cm H_2_O)	ECMO BF (l/min)	Gas Flow (L/min)	In-hospital Survival
1	79	male	10	10	12	18	10	8	3.5	6	NS
2	80	male	12	7	18	20	8	8	3.2	5	S
3	68	male	11	11	23	17	8	12	4	5	NS
4	78	male	14	10	23	16	10	8	3.2	6	S
5	75	male	12	59	60	13	10	8	4	5.5	NS
6	78	male	12	17	25	18	8	10	3.1	5	S
7	66	male	14	7	8	17	8	12	3.6	5	S
8	86	male	12	17	30	8	8	12	3	8	NS
9	59	male	15	8	13	22	8	10	3.5	6	NS
10	84	male	9	4	8	21	8	12	3.5	6	S
Mean ± SD	75 ± 9	10 M	12 ± 2	15 ± 16	22 ± 15	17 ± 4	9 ± 1	10 ± 2	3.5 ± 0.4	5.8(5.0–6.0)^*^	5 S/5NS

SOFA: Sequential organ failure assessment; ECMO: extracorporeal membrane oxygenation; MV: mechanical ventilation; Cst: Static lung compliance; PEEP: Positive end expiratory pressure; PSV: pressure support ventilation; BF: blood flow; NS, non-survival; S, survival

*:median (interquartile range)

In order to increase the level of PaCO_2_ by 5–10 mmHg under PSV, the ECMO GF was decreased from baseline 5.8 (5.0–6.0) L/min to 2.9 (2.5–3.0) L/min in the high-CO_2_ phase (*p* = 0.0020). Arterial blood gas analysis showed that the patient’s PaCO_2_ increased from 36 (36–37) mmHg at baseline to 42 (41–43) mmHg in the high-CO_2_ phase (*p* = 0.0020) (Table 2).

**Table 2:** Variations in the Breathing Pattern during Decrease of Extracorporeal Membrane Oxygenation Support in COVID-19 Patients Undergoing Pressure Support

Characteristic	Baseline	High-CO_2_	*P* value
ECMO GF (l/min)	5.8(5.0–6.0)	2.9(2.5-3.0)	0.0020
PMI(cmH_2_O)	3.0 ± 1.4	6.5 ± 2.1	< 0.0001
ΔPOCC(cmH_2_O)	5.6(4.7-8.0)	11.1(8.5–13.1)	0.0020
RR(bpm)	13 ± 2	15 ± 2	0.0266
MVe (l/min)	3.6 ± 1.3	5.3 ± 1.5	< 0.0001
Vt (ml/kg)	4.1 ± 1.2	5.3 ± 1.5	0.0003
pH	7.42 ± 0.06	7.41 ± 0.05	0.1297
PaCO_2_ (mmHg)	36(36–37)	42(41–43)	0.0020
PaO_2_ (mmHg)	94(87–109)	93(85–106)	0.5742
Arterial Lactate(mmol/L)	1.6 ± 0.4	1.8 ± 0.5	0.1488
HR(bpm)	82 ± 14	89 ± 14	0.0078
MAP(mmHg)	88 ± 11	92 ± 10	0.1776
NE(ug/kg/min)	0.09 ± 0.10	0.09 ± 0.10	> 0.99
Fentanyl (ug/h)	60(56–75)	60(56–75)	> 0.99
Propofol (mg/h)	60(30–55)	60(30–55)	> 0.99
Midazolam(mg/h)	3 ± 2	3 ± 2	> 0.99

Values are given as mean + standard deviation or median (interquartile range)

ECMO: Extracorporeal Membrane Oxygenation; GF: gas flow; PMI: pressure muscle index; ΔPOCC: the airway pressure swing during the occlusion; RR: respiratory rate; MVe: minute volume expiration; Vt: tidal volume; PaCO_2_: partial pressure of carbon dioxide in arterial blood gas; PaO_2_: Oxygen partial pressure of arterial blood gas; HR: heart rate; MAP: mean arterial pressure; NE: norepinephrine

**Fig. 2:**
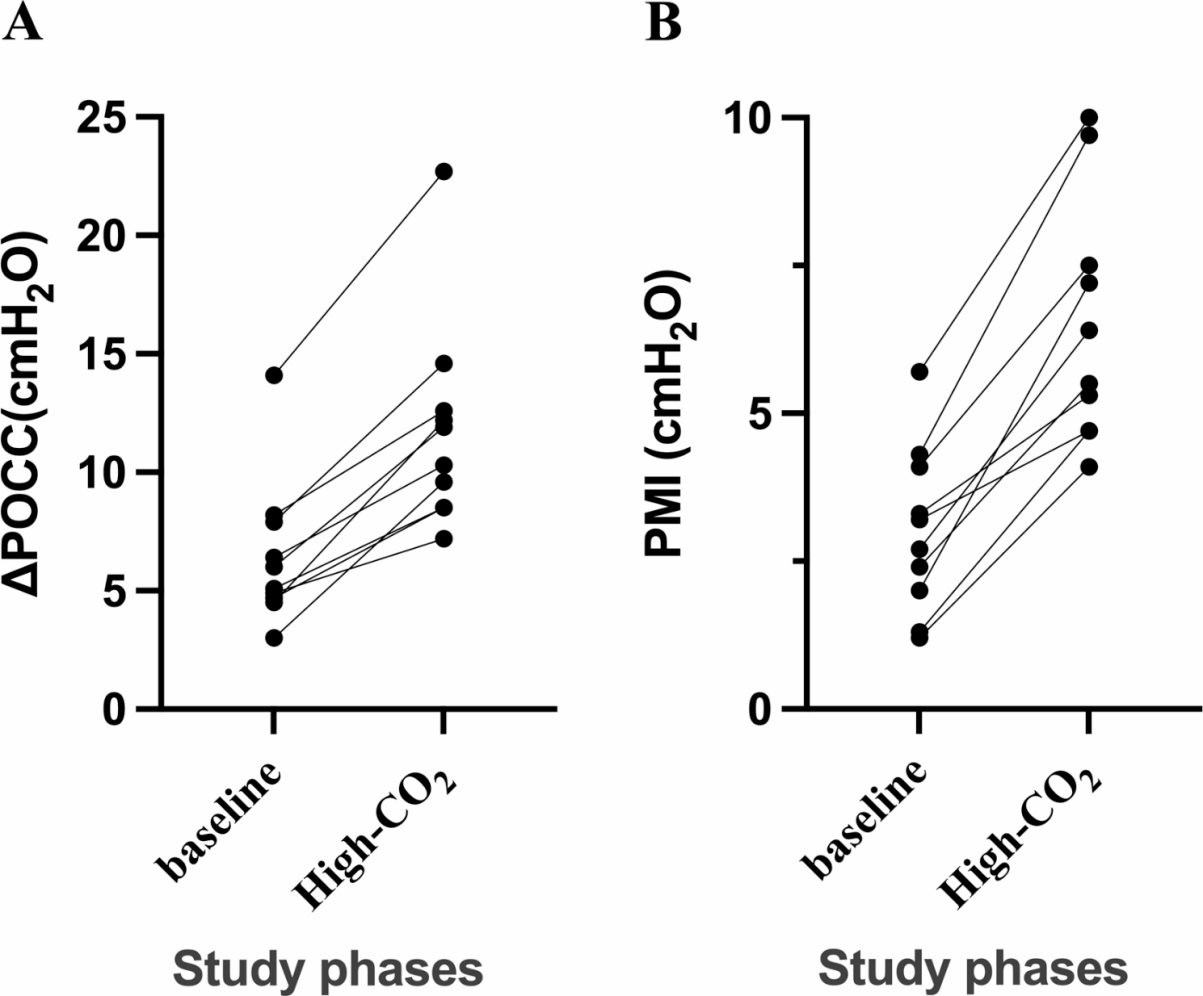
Difference in ΔPOCC and PMI between the two phases. ΔPOCC, airway pressure swing during occlusion; PMI, pressure muscle index

After PaCO_2_ was increased by 5– 10 mmHg, there was a significant increase in ΔPOCC [from 5.6 (4.7–8.0) to 11.1 (8.5–13.1) cm H_2_O, *p* = 0.0020] and PMI [from 3.0 ± 1.4 to 6.5 ± 2.1 cm H_2_O, *p* < 0.0001] (Table 2; Fig. 2). Meanwhile, increased inspiratory effort determined by elevated PaCO_2_ levels led to enhancement of tidal volume from 4.1 ± 1.2 mL/kg to 5.3 ± 1.5 mL/kg (*p* = 0.0003) and respiratory rate from 13 ± 2 bpm to 15 ± 2 bpm (*p* = 0.0266) (Table 2).

**Fig. 3:**
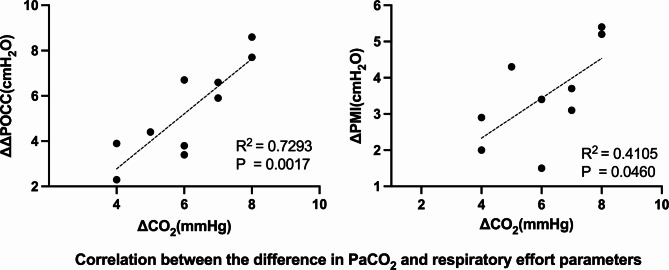
Correlation between the difference in PaCO_2_ and respiratory effort parameters. ΔPOCC, airway pressure swing during occlusion; PMI, pressure muscle index; ΔPaCO_2_, the value of PaCO_2_ in the High PaCO_2_ phase minus the base PaCO_2_ value; ΔΔPOCC, the value of ΔPOCC in the High PaCO_2_ phase minus the base ΔPOCC value; ΔPMI, the value of PMI in the High PaCO_2_ phase minus the base PMI value

In addition, the increase in PaCO_2_ was linearly correlated with changes in ΔPOCC and PMI (R^2^ = 0.7293, *p* = 0.0003 and R^2^ = 0.4105, *p* = 0.0460, respectively) (Fig. 3).

However, the HR of the high-CO_2_ phase was higher than that of the baseline phase (89 ± 14 vs. 82 ± 14 bpm, *p* = 0.0078), meanwhile, with the same norepinephrine dose, there was no statistically significant difference in mean arterial pressure between the two phases.

## Discussion

Herein, we analyzed the effect of PaCO_2_ on the respiratory drive in patients with COVID-19 who had undergone PSV with ECMO. Higher PaCO_2_ levels were associated with a greater respiratory drive.

Previously, it was shown that in removal of CO_2_ by ECMO could induce apnea in healthy and injured animal models [13]. Moreover, two studies showed similar results in patients on vv-ECMO. Marcolin et al. showed that in spontaneously breathing patients with acute respiratory failure, increased ECMO GF critically affected minute ventilation [14]; Moreover, Karagiannidis et al. showed an increase in diaphragm electrical activity (Edi) due to reduction in ECMO GF [15]. Mauri et al. showed that reducing CO_2_ removal by ECMO increased the first 100 min of inspiration against an occluded airway (P_0.1_) and ΔPOCC in patients who had undergone ECMO and recovering from ARDS through PSV and neurally adjusted ventilatory assist [9]. At the same time, the work of breathing, tidal volume, minute ventilation, and airway pressure also increased with the reduction in CO_2_ removal by ECMO. A recent study on the acute exacerbation of chronic obstructive pulmonary disease showed that the respiratory drive (assessed by Edi) increased in the unsuccessful and successful weaning phases during stepwise weaning from venovenous extracorporeal CO_2_ removal [16].

We observed the effect of PaCO_2_ on the respiratory effort of patients with COVID-19 who had undergone PSV with ECMO. Compared with the target value of PaCO_2_ at 35–40 mmHg, a higher PaCO_2_ (> 40 mmHg) was accompanied by a stronger respiratory effort.

### Appropriate PaCO_2_ target

Based on our results, in patients with COVID-19 undergoing PSV with ECMO, an increase in PaCO2 level causes an enhanced respiratory effort. Thus, excessive respiratory effort may be able to be reduced in these patients by decreasing PaCO_2_. The benefits may be as follows: (1) Reducing the patient’s high respiratory effort, thus reducing SILI caused by trans-pulmonary pressure exceeding protective limits. (2) Appropriate respiratory drive is beneficial for maintaining patients with COVID-19 on ECMO in a state of spontaneous breathing with adequate choking capacity, which could help improve sputum drainage, promote lung aeration, improve lung compliance, and accelerate the improvement of COVID-19 pneumonia.

A linear correlation was also observed between the increase in PaCO_2_ and the elevation of ΔPOCC (R^2^ = 0.7293, *p* = 0.0003). The same phenomenon was also observed between PaCO_2_ and PMI (R^2^ = 0.4105, *p* = 0.0460). This may also confirm the role of PaCO_2_ levels in the regulation of respiratory effort in patients with COVID-19 on ECMO, providing a method for titrating the respiratory effort in these patients.

However, proper respiratory drive is necessary to maintain pulmonary aeration. In patients on long-term ECMO support, maintaining appropriate lung aeration can promote lung opening and decrease disuse myopathy [17]. Therefore, very low paCO_2_ level may not be necessary, which may cause acid-base disturbances and other pathophysiological conditions.

### Timing of spontaneous breathing

In the early stages of severe ARDS, respiratory drive is often too strong, accompanied by excessive respiratory mechanical power. Therefore, spontaneous breathing in patients with severe ARDS undergoing ECMO was considered dangerous if it was used too early [18]. Furthermore, the general clinical practice suggests that attempts to perform spontaneous breathing in the early stages of severe ARDS are often impossible. A similar phenomenon was observed in this study. In the early days of vv-ECMO support, the patient’s spontaneous respiratory effort was often so strong that muscle relaxants along with analgesics and sedatives were required to control it. Therefore, patients in our study were on MV for 22 ± 15 days and ECMO support for 15 ± 16 days at the time of enrolment.

Based on our results, lower respiratory effort by increasing CO_2_ clearance in patients with COVID-19 on ECMO may also be appropriate in the early stages of the disease.

Therefore, whether lower PaCO_2_ is beneficial for perform spontaneous breathing earlier deserves further investigation.

### Limitations

There were a few limitations to this study: (1) Ten patients were included in the study, similar to previous studies [9, 19]. However, their sample size was relatively small, which may have increased the occurrence of type II errors. (2) The PaCO_2_ alteration lasted only 20 min before the second measurement was taken but this appeared to be sufficient to obtain stable changes in respiratory patterns and circulatory alterations in previous studies [5, 9], thus making it unnecessary to continue the study for a longer period. (3) The enrolled patients were no longer in the early stages of COVID-19 pneumonia, as they were maintained in a spontaneous breathing state. Therefore, our results provide limited guidance for patients in the early stages of COVID-19. (4) A recent study showed that a significant relative decrease in PaCO_2_ within the first 24 h after ECMO initiation is associated with an increased incidence of neurological complications [20]. Unfortunately, data on cerebral perfusion did not be recorded and there were also no relevant neurological complications in the enrolled patients after our study. However, the results of that study suggested that a rapid drop in CO_2_ of more than 50% was dangerous, and a drop of less than 30% did not suggest harm. the CO2 drop in our study was relatively small, with a mean drop of 16%.

## Conclusions

In patients with COVID-19 undergoing PSV with ECMO, an increase of PaCO_2_ could increase the inspiratory effort.

### Electronic supplementary material

Below is the link to the electronic supplementary material.

Supplementary Fig. S1: Graphical representation of PMI and ΔPOCC waveform. (**A**) PMI = the difference between end-inspiratory obstructive plateau pressure and pre-obstructive airway pressure (Paw). (**B**) ΔPOCC = the maximum deviation of Paw from PEEP during each expiratory airway occlusion

## Data Availability

The data generated and analyzed during this study are not publicly available due to the protection for the patients’ privacy but are available from the corresponding author on reasonable request.

## Abbreviations

ECMO: Extracorporeal membrane oxygenation
Edi: Diaphragm electrical activity
etCO_2_: End tidal carbon dioxide
FIO_2_: The fraction of inspired oxygen
GF: Gas flow
HR: Heart rate
NAVA: Neurally adjusted ventilatory assist
PaCO_2_: The partial pressure of carbon dioxide in arterial blood
Paw: Airway pressure
PEEP: Positive end expiratory pressure
PSV: Pressure support ventilation
PMI: Pressure muscle index
ΔPOCC: Airway pressure swing during occlusion
P_0.1_: The first 100ms of inspiration against an occluded airway
ScvO_2_: Superior vena cava oxygen saturation
SILI: Self-inflicted lung injury
SOFA: Sequential organ failure assessment
vv-ECMO: Venovenous ECMO
