# Supplementary figures and images for: The level of partial pressure of carbon dioxide affects respiratory effort in COVID-19 patients undergoing pressure support ventilation with extracorporeal membrane oxygenation
Source: BMC Anesthesiol. 2024 Jan 12;24:23. doi: 10.1186/s12871-023-02382-9 (PMC10785506; doi:10.1186/s12871-023-02382-9)

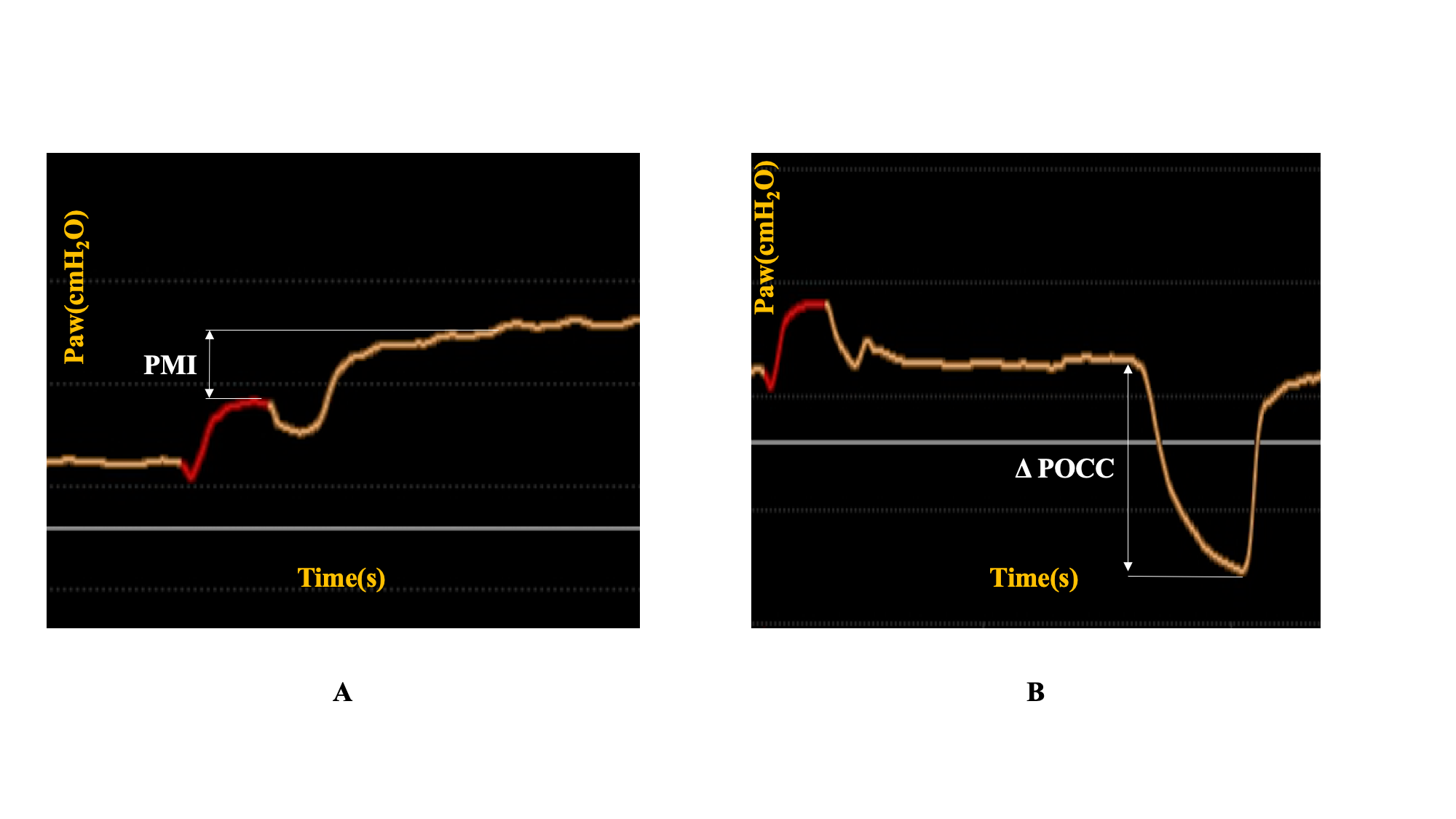

Supplement: Supplementary file 1 — Supplementary Fig. S1: Graphical representation of PMI and ΔPOCC waveform. (A) PMI = the difference between end-inspiratory obstructive plateau pressure and pre-obstructive airway pressure (Paw). (B) ΔPOCC = the maximum deviation of Paw from PEEP during each expiratory airway occlusion [file 12871_2023_2382_MOESM1_ESM.png]
